# Malignant Pleural Mesothelioma Epidemiology in the United States From 2000 to 2016

**DOI:** 10.7759/cureus.14605

**Published:** 2021-04-21

**Authors:** Akesh Thomas, Sajin Karakattu, Jeanette Cagle, Girendra Hoskere

**Affiliations:** 1 Internal Medicine, East Tennessee State University Quillen College of Medicine, Johnson City, USA; 2 Pulmonary and Critical Care Medicine, East Tennessee State University Quillen College of Medicine, Johnson City, USA

**Keywords:** malignant pleural mesothelioma, asbestos, mesothelioma, national cancer database and seer analyses

## Abstract

Introduction

Pleural mesothelioma constitutes about 80% of all mesotheliomas. The peak incidence of malignant mesothelioma estimated using the cancer registries was in early 1990 to 2000 in the United States. The disease is primarily associated with asbestos exposure. The latency period between asbestos exposure and the development of malignant pleural mesothelioma (MPM) can range anywhere from 15 to 60 years. Asbestos exposure was peaked during the industrial revolution and World War II due to military and shipyard exposures. It is often difficult for the pathologist to distinguish different histological subtypes; due to the disease's rarity and the inadequate tissue sample obtained. There is no available data on the difference in epidemiology of different subtypes of MPM. Surveillance Epidemiology and End Results (SEER), cancer incidence data include population-based registries covering approximately 34.6% of the U.S. population. Here in our study, we analyze malignant pleural mesothelioma epidemiology in the United States, emphasizing different histological subtypes.

Methods

SEER data from 2000 to 2016 was used in our study. The primary site of cancer is selected as pleura, and malignant behavior only is selected as the filter. Data were analyzed using the SEER stat program. Overall epidemiology of MPM and epidemiology of epithelioid, fibrous, and biphasic histological subtypes were analyzed separately. We used annual percentage change (APC) to evaluate the trend in the epidemiology of MPM.

Results summary

A total of 11,857 cases of MPM were included in the primary cohort from the SEER 18 registry from 2000 to 2016. The total prevalence of MPM was highest in 2009 and was lowest in 2016. The APC in MPM incidence during this period is -2.0. After removing 5,989 cases with non-specified histology during the same period, the APC for each histological type is -0.7 for fibrous type, 1.8 for epithelioid type, and 2.9 for biphasic type. Out of 17 regional registries included in the study, the greatest statistically significant change in APC was seen in the Hawaiian registry -4.1. In contrast, the lowest statistically significant difference was seen in Seattle (Puget Sound) registry -1.7. The APC in the incidence of MPM among males during the study period was -2.4 while that of females was -0.9. The Iowa registry showed a statistically significant increase in APC of the epithelioid malignant mesothelioma with a statistically insignificant reduction in the overall MPM APC.

Conclusion

The overall incidence of MPM in the United States is declining, while the data showed an increase in the incidence of epithelioid and biphasic histological subtypes. The authors believe that these conflicting results can be attributed to improved histological diagnosis and improved biopsy techniques.

## Introduction

Mesothelioma is a neoplasm originating from the body's mesothelial surfaces, with about 80% of cases being pleural in origin. The United States (U.S.) cancer statistics division of the center for disease control (CDC) estimates the five-year prevalence of mesothelioma to be about 4,562 in the United States [1]. The peak incidence of malignant mesothelioma estimated using the cancer registries was in early 1990 to 2000 in the United States [2], while the peak incidence in England was around the year 2015 [3]. The disease is mostly associated with asbestos exposure; one case per million of the general population is the incidence of non-asbestos-related mesothelioma [4]. Radiation exposure is a significant risk factor in non-asbestos-related cases [5]. The latency period between asbestos exposure and the development of malignant pleural mesothelioma (MPM) can range anywhere from 15 to 60 years [6]. Three different histological subtypes of MPM are typically identified; epithelioid, sarcomatoid, and biphasic [7]. It is often difficult for the pathologist to distinguish different histological subtypes due to the disease's rarity and the inadequate tissue sample obtained. There is no available data on the difference in epidemiology of different histological subtypes of MPM. But, studies suggest that the epithelioid subtype is associated with better survival than the other subtypes [8,9].

Asbestos exposure peaked during the industrial revolution and World War II due to military and shipyard exposures [10]. The mesothelioma hot spots in the Unites States can be traced to communities that lived near industries that used asbestos in abundance, like Manville in New Jersey and Libby in Montana. On the other hand, California has the largest natural asbestos deposit, increasing MPM incidence in the state. Asbestos is the generic name for a group of naturally occurring minerals that contain silicate tetrahedron (SiO4). The arrangement and number of tetrahedra determine the classification of the mineral. Six arrangements are classified as asbestos: chrysotile, actinolite, amosite, anthophyllite, crocidolite, and tremolite. Each of these arrangements has varying chemical and physical properties, but all share carcinogenic risks. Exposure through inhalation and ingestion must occur for asbestos to cause cancer. Other methods of exposure, such as dermatologic contact, are not associated with any carcinogenic risk. Exposures to crocidolite, amosite, and chrysotile are most commonly associated with pleural mesothelioma development [11]. Asbestos in all forms is also associated with the development of pharynx, stomach, and colorectal cancers [12]. The determinants of asbestos toxicity are fiber size, bio persistence, chemical composition, and particle surface characteristics [13]. The proposed mechanisms by which asbestos causes carcinogenesis are direct interaction with cellular chromosomes, generation of reactive oxygen species, and inflammation [14]. The occupational asbestos exposure has been significantly reduced since the 1970s, leading to a steady decline in MPM incidence after the year 2000. The world trade center disaster in 2001caused significant asbestos particle exposure among people living in New York City and those involved in rescue work [15], which can lead to MPM development decades later.

Surveillance, Epidemiology, and End Results (SEER) cancer incidence data include population-based registries covering approximately 34.6% of the U.S. population. Here in our study, we analyze malignant pleural mesothelioma epidemiology in the United States, emphasizing different histological subtypes.

## Materials and methods

SEER data from 2000 to 2016 was used in our study. Data included 18 regional registries in SEER. Cancer's primary site was selected as pleura, and malignant behavior only was chosen as the filter in SEER software. We excluded age zero and unknown age from the data. For the histological classification, we excluded the unknown histological type. Data were analyzed using the SEER stat program [16]. Overall epidemiology of MPM and epidemiology of epithelioid, fibrous, and biphasic histological subtypes were studied separately. Data were analyzed independently for gender, ethnicity, age groups of 45-49, 50-54, 55-59, 60-64, 65-69, 70-74, 75-79, and the 17 different regions. We used annual percentage change (APC) to evaluate the trend in the epidemiology of MPM. The APC was calculated using the incidence rate per 100,000 of the U.S. population age-adjusted to the 2000 U.S. standard population (19 age groups - Census P25-1130). The percentage change is calculated using one year for each endpoint. A 95% confidence interval is included with all the calculated results for assessing the significance.

## Results

**Figure 1:**
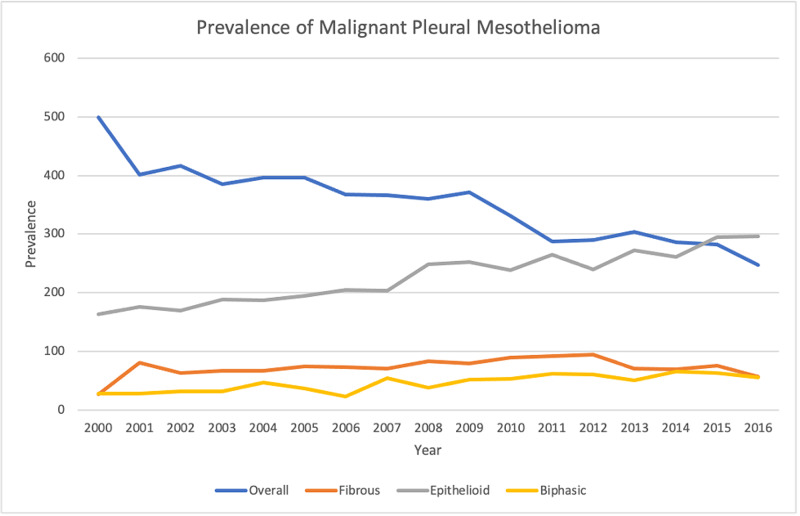
Prevalence of malignant pleural mesothelioma.

**Figure 2:**
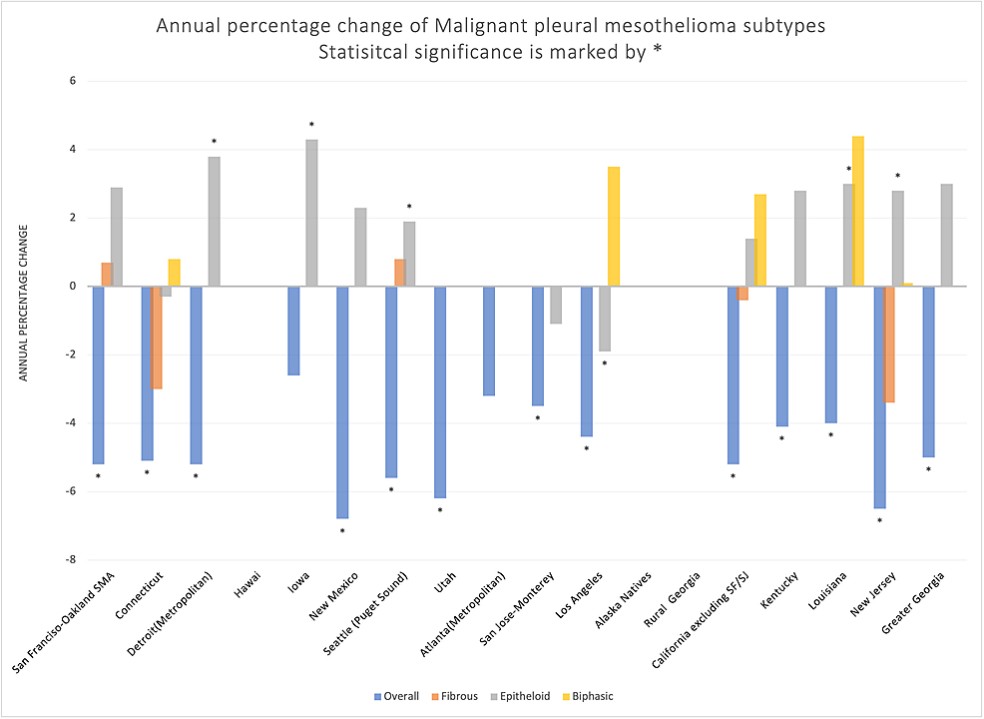
APC of MPM subtypes. APC: annual percentage change; MPM: malignant pleural mesothelioma. *Statistical significance.

**Figure 3:**
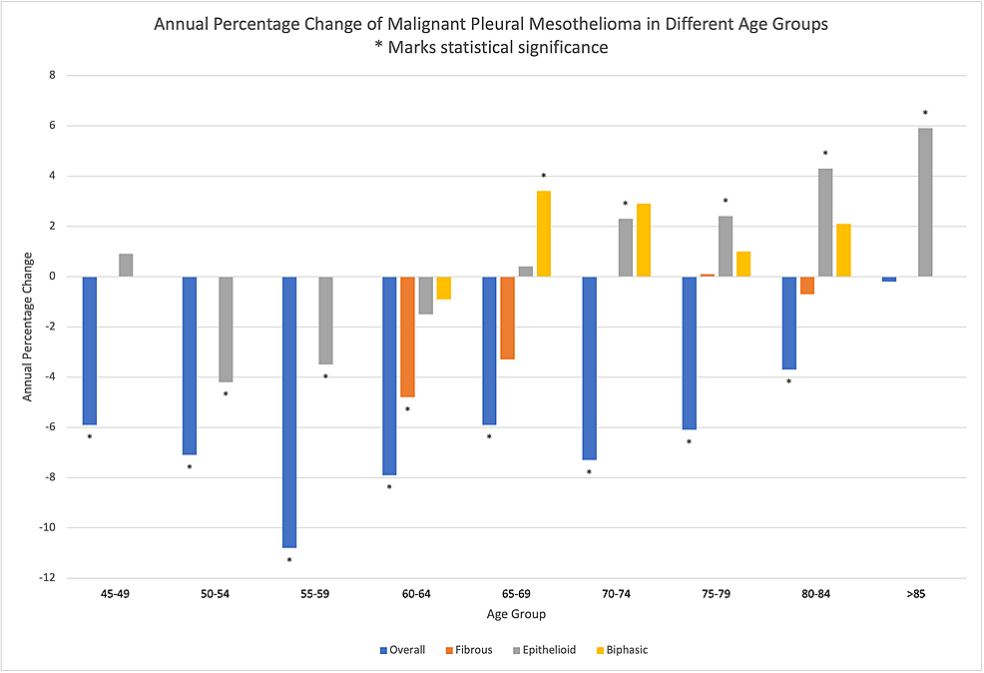
APC of MPM in different age groups. APC: annual percentage change; MPM: malignant pleural mesothelioma. *Statistical significance.

A total of 11,857 cases of MPM were included in the primary cohort from the SEER 18 registry from 2000 to 2016. The total prevalence of MPM was highest in 2000 and was lowest in 2016. The overall prevalence of MPM decreased during the study period, while the epithelioid MPM prevalence increased during the same period (Figure 1). The overall annual percentage change (APC) in MPM incidence during this period is -2.0, 95% CI [-2.4, -1.5]. The APC for each histological type after removing 5,989 cases with non-specified histology during the same period was -0.7[-3.1,1.7] for fibrous type, 1.8 [1.2, 2.3] for epithelioid type, and 2.9 [0.8, 5.1] for biphasic type. Out of 17 regional registries included in the study, the greatest statistically significant change in APC was seen in the Hawaiian registry -4.1 [-7.6, -0.4] while the lowest statistically significant difference was seen in Seattle (Puget Sound) registry -1.7 [-2.7, -0.6]. A significant change in APC was seen in San Francisco-Oakland, Connecticut, Seattle, Los Angeles, California, and New Jersey registries. In contrast, Detroit(metropolitan), Iowa, New Mexico, Utah, Atlanta, San Jose-Monterey, Kentucky, Louisiana, and Greater Georgia registries did not show a significant change in APC (Figure 2). Detroit metropolitan region, Seattle (Puget Sound), Louisiana, and New Jersey, registries showed a statistically significant increase in the epithelioid variant of MPM while the overall APC for MPM in the region still showed a statistically significant decrease during the study period. Contrary to this, the Iowa registry showed a statistically significant increase in APC of the epithelioid malignant mesothelioma with a statistically insignificant reduction in the APC overall for MPM. Statistical calculations were not possible in region-wise APC for fibrous and biphasic MPM in most regions due to the inadequate number of patients. The APC in the incidence of MPM among males during the study period was -2.4 [-3, -1.8] while that of females was -0.9 [-1.5, -0.2]. Of all the age groups from 45 to 85 (in multiples of 5), the APC for overall malignant mesothelioma showed a statistically significant decrease in all the groups. Fibrous MPM showed a statistically significant reduction in the age group of 60-64 only. Epithelioid MPM showed a statistically significant reduction in APC among age groups 50-54 and 55-59, while it showed a statistically significant increase in age groups 70-74,75-79, and 80-84. Biphasic MPM showed a statistically significant increase in the age group 65-69 only (Figure 3). Blacks, whites, and Asian/pacific islanders showed a statistically significant decrease in the APC of overall MPM. While whites showed a statistically significant increase in APC for both epithelioid and biphasic MPM, all the other racial subclasses were either statistically insignificant or unable to calculate. (For detailed data, see Tables 1-3 in the appendix).

## Discussion

The analysis of the MPM epidemiology clearly shows a decline in the total prevalence and overall incidence of MPM in the United States. Despite this, there is an increase in the prevalence of epithelioid and biphasic MPM subtypes with a positive APC during the study period. The authors believe this paradoxical statistic results from increased diagnostic accuracy of MPM histological subtypes during the study period. Increased awareness of the histological variants, availability of better tissue samples with increased access to better techniques like video-assisted thoracoscopy, and improved pathological diagnostic techniques might have helped. The challenges that pathologists faced in making an accurate diagnosis of mesothelioma is well reported in the literature [17]. Magner and McDonald in 1972 suggested the association of histological types of MPM differently to asbestos exposure (with mixed cell type more likely associated with asbestos exposure) [18], but this hypothesis was later disproved on further analysis [19]. Most regional MPM statistics followed the national trend as expected, with a negative APC for overall MPM incidence with either positive or negative APC for the epithelioid subtype. An exception is the Iowa registry, which showed a statistically significant positive APC for the incidence of epithelioid MPM with a statistically non-significant negative APC for overall MPM incidence. Whether the Iowa registry is of concern needs further detailed investigation. Among all age groups from 45 to 85, there is a significant decline in the overall incidence of MPM. A significant increase in the epithelioid MPM is seen in persons older than 70. During the study period, those older than 70 are members of the population that lived through the second world war and the industrial revolution, with obviously more asbestos exposure than the younger people during the study period. Is this a mere coincidence or a suggestion of a correlation between asbestos exposure and epithelioid type MPM (than other histological types) needs further detailed analysis. Besides, there are suggestions that the mesothelioma in young people (age < 40) may be less related to asbestos exposure than those seen in the older population [20].

The accurate diagnosis of MPM histological subtype needs special training for the pathologist [21]. The most common subtype epithelioid MPM consists of a heterogeneous group of histopathology, including solid, tubulopapillary, trabecular, micropapillary, deciduoid, and pleomorphic [22]. Even within these variants of epithelioid subtype, the prognosis varies significantly [23,24], which signifies the importance of making an accurate histological diagnosis important. We recommend that all the MPM diagnoses should be confirmed for the histology in a center with expertise in the pathological diagnosis of MPM.

The authors acknowledge that the study is limited by the basic characteristics of the registry itself. The study's retrospective nature and the lack of information on whether the histopathological diagnosis was made in an expert center are limitations of the study. Deriving a conclusion on the epidemiology of the different histological types of MPM using the registry data can be potentially erroneous due to numerous biases, including selection bias and reporting bias. The lack of data from some of the states with a high incidence of MPM, like Pennsylvania, Florida, and Texas, is another limitation of this study.

## Conclusions

The overall incidence of MPM in the United States is declining, while the data showed an increase in the incidence of epithelioid and biphasic histological subtypes. The authors believe that these conflicting results can be attributed to the improved histological diagnosis and improved biopsy techniques, including video-assisted thoracoscopy. Iowa registry showed a significant increase in epithelioid MPM without a significant decline in overall MPM incidence. There is more decline in MPM incidence in males than females, while the incidence of MPM in males still remains higher than in females.

## Appendices

**Table 1:** Number of newly diagnosed malignant pleural mesothelioma cases per year.

Year	Mesothelioma total	Fibrous mesothelioma	Epithelioid mesothelioma	Biphasic mesothelioma
2000	499	26	163	28
2001	402	81	176	28
2002	417	63	170	32
2003	385	67	188	32
2004	396	67	187	46
2005	396	74	195	36
2006	368	73	205	23
2007	366	71	203	54
2008	360	83	249	38
2009	372	79	252	52
2010	331	89	239	53
2011	288	92	265	62
2012	290	94	240	61
2013	304	71	272	51
2014	286	69	261	66
2015	282	76	295	63
2016	247	57	296	55

**Table 2:** Overall MPM annual percentage change in different registries. *Statistically significant. ~Cannot be calculated. MPM: malignant pleural mesothelioma.

Region	Rate/trend	Lower CI	Upper CI
San Francisco-Oakland SMSA - 2000+	-2.1*	-3.8	-0.4
Connecticut - 2000+	-2.5*	-4.6	-0.3
Detroit (Metropolitan) - 2000+	-0.9	-3	1.3
Hawaii - 2000+	-4.1*	-7.6	-0.4
Iowa - 2000+	1.4	-0.9	3.7
New Mexico - 2000+	-2.3	-5.3	0.8
Seattle (Puget Sound) - 2000+	-1.7*	-2.7	-0.6
Utah - 2000+	-2.3	-5	0.4
Atlanta (Metropolitan) - 2000+	-0.8	-3.2	1.7
San Jose-Monterey - 2000+	-1.5	-3.7	0.8
Los Angeles - 2000+	-2.6*	-3.8	-1.5
Rural Georgia - 2000+	~	~	~
California excluding SF/SJM/LA - 2000+	-2.2*	-3.4	-1.1
Kentucky - 2000+	-0.4	-2	1.2
Louisiana - 2000+	-0.7	-1.8	0.4
New Jersey - 2000+	-2.8*	-3.8	-1.9
Greater Georgia - 2000+	-1.7	-3.7	0.3

**Table 3:** Annual percentage change of histological subtypes of MPM. *Statistically significant. ~Cannot be calculated. MPM: malignant pleural mesothelioma.

Region	Fibrous	Epithelioid	Biphasic	Total
San Francisco-Oakland SMSA - 2000+	0.7	2.9	~	-5.2*
Connecticut - 2000+	-3	-0.3	0.8	-5.1*
Detroit (Metropolitan) - 2000+	~	3.8*	~	-5.2*
Hawaii - 2000+	~	~	~	~
Iowa - 2000+	~	4.3	~	-2.6
New Mexico - 2000+	~	2.3	~	-6.8*
Seattle (Puget Sound) - 2000+	0.8	1.9*	~	-5.6*
Utah - 2000+	~	~	~	-6.2*
Atlanta (Metropolitan) - 2000+	~	~	~	-3.2
San Jose-Monterey - 2000+	~	-1.1	~	-3.5*
Los Angeles - 2000+	~	-1.9*	3.5	-4.4*
Rural Georgia - 2000+	~	~	~	~
California excluding SF/SJM/LA - 2000+	-0.4	1.4	2.7	-5.2*
Kentucky - 2000+	~	2.8	~	-4.1*
Louisiana - 2000+	~	3.0*	4.4	-4.0*
New Jersey - 2000+	-3.4	2.8*	0.1	-6.5*
Greater Georgia - 2000+	~	3	~	-5.0*
